# Percutaneous Mitral Valve Repair with the Edge-to-Edge Technique: Case Series of First Iranian Experience

**Published:** 2014-01-12

**Authors:** Seyed Ebrahim Kassaian, Arsha Karbassi, Mohammad Sahebjam, Hassan Aghajani, Ahmad Amin, Niloufar Ahmadbeigi, Kyomars Abbasi, Abbas Salehiomran, Hamidreza Poorhosseini, Mojtaba Salarifar

**Affiliations:** 1 *Tehran Heart Center, Tehran University of Medical Sciences, Tehran, Iran.*; 2 *Rajaie Cardiovascular, Medical and Research Center, Iran University of Medical Sciences, Tehran, Iran.*

**Keywords:** *Mitral valve* • *Echocardiography* • *Iran* • *Cardiac catheterization*

## Abstract

Mitral regurgitation (MR) is a common valvular lesion in the general population with considerable impact on mortality and morbidity. The MitraClip System (Abbot Laboratories, Abbot Park, IL, USA) is a novel percutaneous approach for treating MR which involves mechanical edge-to-edge coaptation of the mitral leaflets. We present our initial experience with the MitraClip System in 5 patients. In our series, the cause of MR was both degenerative and functional. Two patients received two MitraClips due to unsatisfactory results after the implantation of the first clip. Acute procedural success was seen in 4 patients. Blood transfusion was required for 2 patients. All the patients, except one, reported improvement in functional status during a 2-month follow-up period. Our initial experience with MitraClip implantation indicates that the technique seems feasible and promising with acceptable results and that it could be offered to a broader group of patients in the near future.

## Introduction

Mitral regurgitation (MR) is one of the most commonly encountered valvular lesions, with at least moderate regurgitation being present in 6.4% of the general population above 65 years.^1^ Moderate-severe MR is present in 15-30% of patients with congestive heart failure and up to 12% of patients within one month after myocardial infarction. The severity of MR is positively correlated with the subsequent development of heart failure and death.^2^ Since even asymptomatic patients with severe MR have higher rates of death, heart failure, and atrial fibrillation, consideration of valve repair is clinically warranted in these patients. Before the advent of percutaneous mitral repair procedure, surgical mitral valve repair or replacement was the sole option for treating MR.^3^ The desire for less invasive approaches, coupled with the fact that a significant proportion of patients (especially, elderly persons and those with significant comorbidities or severe left ventricular dysfunction) is not referred for surgery, has driven the development of percutaneous mitral repair devices.^4^

The MitraClip System is a novel percutaneous approach for treating MR which involves mechanical edge-to-edge coaptation of the mitral leaflets. Mitral valve repair with the use of a surgical approach to create a double-orifice valve without annuloplasty was first performed by Alfieri in 1991.^5^^, ^^6^ Percutaneous mitral repair based on this surgical technique has been developed by the use of a clip rather than suture to secure the mitral leaflets.^7^ The first human implant of the MitraClip was performed in 2003.^8^ The MitraClip device obtained "CE Mark of Approval" in 2008 and is now commercially available in approximately 30 countries, with more than 9,000 patients having been treated with it to date.^9^

**Figure 1:**
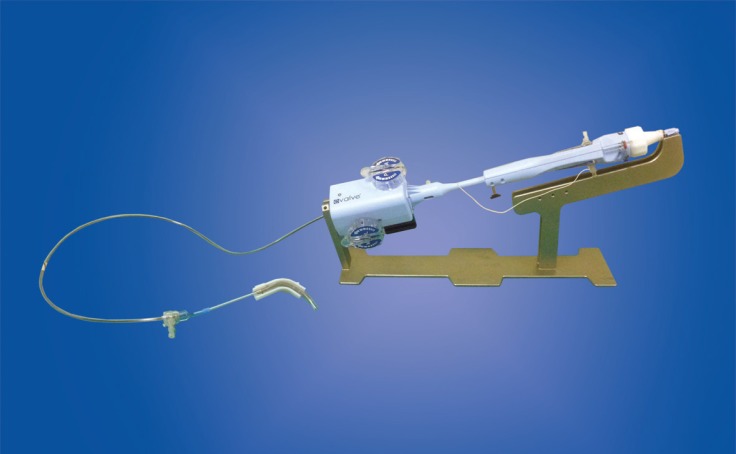
MitraClip delivery system

The system consists of a steerable guide catheter and a clip delivery system (CDS), which includes the detachable clip. There is also a stabilizer that keeps the system precisely in position. The clip is Dacron-covered with two arms that are opened and closed by a control mechanism on the CDS. The tip of the guide catheter is delivered to the left atrium using the trans-septal approach over a guide wire and tapered dilator. The guide catheter is 24 Fr proximally, and tapers to 22 Fr at the point where it crosses the atrial septum. A steering knob on the proximal end of the guide catheter, marked as +/-, allows for the flexion of the distal guide catheter tip (Figure 1).^10^

At Tehran Heart Center, a Tertiary University Hospital affiliated to Tehran University of Medical Sciences, we manage patients with MR requiring mitral valve replacement or surgical repair as a treatment modality. However, some of these patients refuse surgery or are deemed high risk for surgery. In order to provide this group of patients with appropriate treatment, a group of interventional cardiologists and an echocardiologist from this center trained for the MitraClip procedure. 

## Methods

Patients were evaluated by a panel comprising 3 interventional cardiologists, one echocardiologist, one cardiac anesthesiologist, and 2 cardiac surgeons at a dedicated clinic for the MitraClip candidates. Patients had a standard diagnostic work-up, including physical examination, functional capacity assessment [New York Heart Association (NYHA) class], electrocardiography, blood tests, transthoracic echocardiography (TTE) and transesophageal echocardiography (TEE), and coronary artery disease evaluation. Patients were selected if they met class I indication for intervention according to 2008 American College of Cardiology / American Heart Association (ACC/AHA) guidelines.^11^

MR was graded according to the criteria of The American Society of Echocardiography (ASE) guidelines by the use of quantitative and qualitative criteria.^12^ Key anatomic inclusion criteria included those used in the Endovascular Valve Edge-to-Edge Repair Study (EVEREST) trials: MR originating from the central 2/3 of the valve, mitral valve area (MVA) ≥ 4cm^2 ^, a coaptation length ≥ 2 mm and a coaptation depth ≤ 11 mm for patients with functional MR, and a flail gap < 10 mm and flail width < 15 mm for patients with degenerative MR.^13^^-^^15^ All pre-procedural echocardiographic examinations of the selected patients were sent to a core echocardiography laboratory in Belgium for review.

All the procedures were performed in the catheterization laboratory with specific sterile precautions. 

The procedure was performed under general anesthesia. Prophylactic antibiotics were given before the procedure, and all the patients were pretreated with Aspirin and Clopidogrel. 

A 14-Fr sheath was placed in the right femoral vein for trans-septal access. At a later stage of the procedure, the steerable guide catheter would replace it. Initially, a trans-septal needle and the trans-septal sheath were advanced into the right atrium. Trans-septal puncture should be placed relatively posterior and high in the fossa ovalis. This high puncture allows adequate working space and distance above the mitral leaflets (3.5-4 cm above the plane of the mitral annulus). Via TEE short-axis view at the base of the heart, the position of the potential puncture site was evaluated prior to needle advancement. As a measure of safety, the puncture point should never be in the septum secundum. Intravenous Heparin was given (50-70 units/kg) to achieve ACT ≥ 250 sec after successful trans-septal puncture.^10^

**Figure 2:**
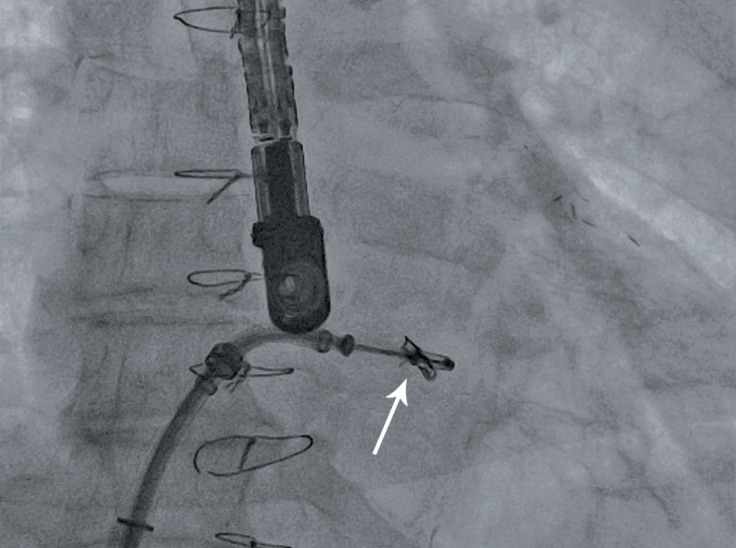
Fluoroscopic image, showing the clip with open arms (arrow)

**Figure 3:**
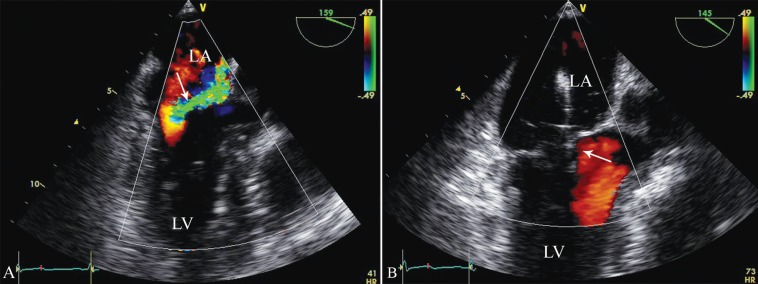
Echocardiographic view, showing severe mitral regurgitation in color Doppler (arrow) (A), which is reduced to mild mitral regurgitation (arrow) after device implantation (B)

LA, Left atrium; LV, Left ventricle

A 0.035-inch Superstiff exchange length guide wire was advanced through the trans-septal catheter to the upper pulmonary vein. The trans-septal catheter was then removed and exchanged with the guide catheter. The MitraClip attached to the CDS was then advanced through the guide catheter into the left atrium with the help of multiplane TEE. The device was oriented appropriately over the mitral valve. Once properly oriented, the clip was advanced to the left ventricle (LV) (Figure 2) and the CDS was pulled back and the leaflets were grasped by dropping the grippers. After confirmation of adequate grasping of the leaflets, the arms were closed and reduction in MR was assessed. If the reduction in MR was adequate, the clip was deployed (Figure 3). Acute procedural success was defined as a post-procedure reduction of MR to ≤ 2+. In addition to the assessment of MR, mitral valve gradients were checked periodically throughout the procedure to ensure that there was no iatrogenic mitral stenosis.^10^

A figure-of-eight suture was used to obtain hemostasis at the 24-F access site. The patients were transferred to the Coronary Care Unit. Aspirin (80 mg daily, lifelong) and Clopidogrel (75 mg daily for 3 months) were prescribed. Before discharge, all the patients underwent TTE to assess the position of the clip and residual MR. Endocarditis prophylaxis was advised. After discharge, the patients were followed up monthly for the first 2 months and then bimonthly at the outpatient clinic.

## Results

**Figure 4:**
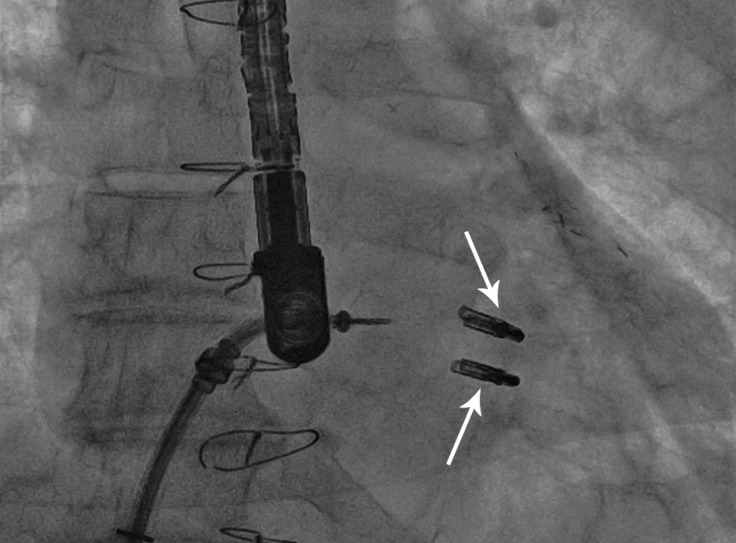
Fluoroscopic image, showing two clips (arrows) implanted in order to reduce mitral regurgitation

Tables 1 and 2 show the clinical and echocardiographic data of our patients. Two patients received two MitraClips due to unsatisfactory results after the implantation of the first clip (Figure 4). Mild functional mitral stenosis was seen in both of these patients. Acute procedural success was achieved in all the patients, with the exception of one patient, in whom MR was reduced to moderate despite intensive effort and implantation of two clips. There was no further space in order to deploy a third device. No clip detachment occurred. Two patients required blood transfusion; both of these patients had received two clips with a longer procedural time. The hemoglobin drop in the first patient was due to hematoma formation at the puncture site and one unit of packed blood cell was infused. The second patient developed retro-peritoneal hematoma, so surgical consultation was done and two units of packed blood cell were infused. No other intervention was required and the patient was discharged home in good medical condition. No other in-hospital morbidity happened. Post-procedural hospital stay was 2 days in all the patients, with the exception of the patient who developed retroperitoneal hematoma and was admitted for 5 days. All the patients reported clinical improvement in functional status at one and 2-months' follow-ups, with the exception of the patient who had residual moderate MR accompanied by functional mitral stenosis. The latter complained of dyspnea (NYHA class II-III) and was referred for mitral valve replacement.

**Table 1:** Baseline characteristics

	Patient 1	Patient 2	Patient 3	Patient 4	Patient 5
Age (y)	35	55	83	62	58
Gender	Male	Male	Male	Male	Male
LVEF (%)	60	55	55	60	60
LVEDV/LVESV (cc)	97/37	114/52	99/45	110/37	67/28
NYHA Class	II-III	II	II	II	II
CAD	-	+	+	+	+
Previous PCI	-	-	-	+	-
Previous CABG	-	+	+	-	-
Diabetes	-	-	-	-	+
Atrial Fibrillation	-	-	-	-	+

LVEF, Left ventricular ejection fraction; LVEDV, Left ventricular end-diastolic volume; LVESV, Left ventricular end-systolic volume; NYHA Class, New York Heart Association classification; CAD, Coronary artery disease; PCI, Percutaneous coronary intervention; CABG, Coronary artery bypass graft

**Table 2:** Mitral regurgitation characteristics at baseline and post- MitraClip implantation

	Type of MR	Baseline MR Severity	Number of MitraClips	Post-MitraClip MR Severity	MVA (cm^2^)	Mean gradient (mm Hg)	MR Severity
Patient 1	D	Severe	I	Mild	2.34	4.0	Mild
Patient 2	F/D	Severe	I	Mild	2.10	3.1	Mild
Patient 3	D	Severe	II	Mild to Moderate	1.74	5.2	Mild to Moderate
Patient 4	D	Severe	II	Moderate	1.77	5.4	Moderate
Patient 5	F	Moderate to Severe	I	Mild	2.44	4.1	Mild

D, Degenerative; F, Functional; MVA, Mitral valve area (measured by pressure half-time)

## Discussion

The MitraClip System was evaluated in EVEREST phase I and II clinical trials.^13^ Data evaluating the safety and mid-term durability of this device were first reported by Feldman et al.^14^ The primary success rate was 74%, with freedom from death and surgery of 90.1% and 76.3%, respectively, at a medium follow-up of 3.2 years. These encouraging results and low complication rates confirm the safety of this procedure.

EVEREST II was a randomized controlled trial evaluating the safety and efficacy of percutaneous mitral valve repair versus surgical repair or replacement in 279 subjects. Eligible patients were prospectively randomized to MitraClip therapy or mitral valve surgery in a 2:1 ratio. At 12 months, the rate of primary efficacy end-point (composite end-point consisting of death, mitral valve surgery/reoperation, or 3+/4+ MR) was lower for the MitraClip group compared to the surgical group (55% vs. 73%); however, the MitraClip group met criteria for non-inferiority (p value = 0.007). This difference was largely driven by a higher mitral valve surgery/ re-operation rate in the MitraClip group (20% vs. 2%; p value < 0.001). Whereas there was no difference between the two groups in terms of overall mortality and residual MR, the MitraClip was superior with regard to the safety end-point consisting of major adverse events at 30 days (15% vs. 48%; p value < 0.001). This difference was driven by a significantly higher incidence of bleeding requiring transfusion of at least two units of pack red blood cells in the surgical group. Both groups showed significant improvement in NYHA functional class and reduction in LV volumes. The results of the EVEREST II trial allow the conclusion that percutaneous mitral repair provides increased safety, while surgery provides more complete reduction in MR.^15^

In Europe, the MitraClip System has been available for clinical use outside specified study protocols. As a result, European centers have predominantly enrolled patients with functional MR, reduced LV function, and high surgical risk. In contrast, the EVEREST trial program had strict morphological inclusion criteria and particularly enrolled patients with degenerative MR, preserved ejection fraction (EF), and low surgical risk,^14^^-^^18^ (Table 3). In the Middle East, the procedure has been in use in recent years. In Turkey, MitraClip implantation began in late 2010 and up to now 47 patients have undergone this procedure.^19^

Our patients were selected according to the EVEREST trial inclusion criteria. The patients were younger than those in other studies with preserved left ventricular ejection fraction (LVEF). Type of MR was both degenerative and functional, which is consistent with other studies. Acute procedural success was seen in all of our patients except in one patient who had degenerative MR. In our experience, 2 patients required blood transfusion due to hematoma formation. It could be proposed that in order to preclude hematoma formation, Clopidogrel should be started after device deployment in order to prevent overt antiplatelet dysfunction during the procedure.

Echocardiographic studies on patients with the MitraClip have shown reverse LV remodeling as evidenced by a decrease in LV dimensions. Follow-up studies at 3-12 months have documented a significant reduction in LV end-diastolic and end-systolic volumes as a result of the favorable effect of chronic LV unloading.^20^^-^^22^ LVEF remained unchanged in the majority of cohorts, although a significant increase was observed in studies with the lowest baseline EF.^20^^, ^^21^ Mitral valve area tends to decrease by 1.4-2.4 cm^2^, whereas trans-mitral pressure gradient increases by 1.3-2.4 mmHg after MitraClip implantation.^20^^, ^^21^^, ^^23^

**Table 3:** Comparison of published series of patients treated with the MitraClip

	Feldman^14^	**Feldman** ^15^	**Tamburino** ^17^	**Treede** ^18^
Number of Patients	107	184	31	202
				
EVEREST Eligibility	100%	100%	100%	Not Recorded
				
Degenerative/Functional MR	79%/21%	73%/27%	42%/58%	27%/73%
				
Mean Age (y)	71	67	71	75
				
Baseline LVEF	62%	60%	42%	44%
				
Baseline NYHA III/IV	46%	52%	87%	98%
				
Follow-Up NYHA III/IV (Follow-Up Duration)	8% (12-Month)	2% (12-Month)	0 (30-day)	34% (12-month)
				
APS	74%	Not Recorded	97%	92%
				
MR ≤ 2+ at Follow-Up (Follow-Up Duration)	66% (12-month)	82% (12-Month)	94% (30-day)	89% (12-month)
				
Procedural Mortality	0	Not Recorded	0	Not Recorded

APS, Acute procedural success; MR, Mitral regurgitation

Overall, serious life-threatening or fatal complications related to the MitraClip procedure are exceedingly rare. Procedural mortality is also very low, and the rates of major clinical complications such as stroke, myocardial infarction, acute renal failure, and septicemia are below 5%. Among minor complications, the most common was access site bleeding or groin hematoma. Clip-related complications are rare (< 5%) but potentially deleterious. Complications from trans-septal puncture may include pericardial tamponade with the need for emergency pericardiocentesis and iatrogenic atrial septal defect. Clip-related chordal rupture may result from inadvertent tangling of the device in the sub-valvular apparatus and may give rise to acute MR worsening, requiring emergency circulatory support and bail-out mitral valve surgery.^16^

Operator training is an essential component of the MitraClip procedure and it is also crucial for the candidate center to have sufficient experience in structural heart interventions with good surgical support. The cost of the device is an important issue for our country's health care system; accordingly, MitraClip cases should be carefully selected from among high-risk patients denied by surgeons, because surgery is still the first choice therapy for patients with severe MR.

Based on the initial experience with MitraClip implantation in our center, the technique seems to be feasible and promising with acceptable results and it will be offered to a wider group of patients in the near future.

## Conclusion

Our initial experience with MitraClip insertion in the first series of Iranian patients indicates that it seems to be an appropriate therapeutic method, especially for patients at high risk in open surgical mitral repair. A more in-depth evaluation, however, requires new prospective studies with larger cohorts of patients.
